# AI in medical education: the moderating role of the chilling effect and STARA awareness

**DOI:** 10.1186/s12909-024-05627-4

**Published:** 2024-06-07

**Authors:** Meijie Wu, Xuefeng Huang, Baona Jiang, Zhihong Li, Yuanyuan Zhang, Bo Gao

**Affiliations:** 1 School of Public Health, Dalian Medical University, Dalian, China; 2 Dalian Medical University, Dalian, China; 3 Department of Psychology, Dalian Medical University, Dalian, China; 4 Department of Chinese Medicine and Physiotherapy Dalian Rehabilitation, Recuperation Center of Joint Logistics, Support Force of PLA, Dalian, China

**Keywords:** Artificial intelligence (AI), Medical education, Chilling effect, STARA awareness, Intention to continue to use

## Abstract

**Background:**

The rapid growth of artificial intelligence (AI) technologies has been driven by the latest advances in computing power. Although, there exists a dearth of research on the application of AI in medical education.

**Methods:**

this study is based on the TAM-ISSM-UTAUT model and introduces STARA awareness and chilling effect as moderating variables. A total of 657 valid questionnaires were collected from students of a medical university in Dalian, China, and data were statistically described using SPSS version 26, Amos 3.0 software was used to validate the research model, as well as moderated effects analysis using Process (3.3.1) software, and Origin (2021) software.

**Results:**

The findings reveal that both information quality and perceived usefulness are pivotal factors that positively influence the willingness to use AI products. It also uncovers the moderating influence of the chilling effect and STARA awareness.

**Conclusions:**

This suggests that enhancing information quality can be a key strategy to encourage the widespread use of AI products. Furthermore, this investigation offers valuable insights into the intersection of medical education and AI use from the standpoint of medical students. This research may prove to be pertinent in shaping the promotion of Medical Education Intelligence in the future.

## Introduction

### Background

The new round of technological revolution continues to stimulate industrial innovation, with the intelligent transformation of the entire industry emerging as the prevailing theme of the times. Artificial intelligence-related applications, as the core hotspot of this transformation, are progressively permeating various fields. In the medical sector, an increasing number of smart products are being integrated to collect daily health data, interpret data, and conduct imaging [1]. The transition of the practice of medicine from the Information Age to the Age of Artificial Intelligence in conjunction with machine learning has led to a transformation in the healthcare industry. The availability of big data has facilitated descriptive, predictive, and prescriptive analysis by data scientists [2], and AI has shown considerable promise in providing medical education.

### Conceptual framework

This study operates within an integration model framework encompassing three research models and two moderator variables, such as the TAM model, UTAUT model, ISSM model, STARA awareness, and chilling effect:

Acceptance and use of information technology is a topic of interest to researchers and practitioners in the fields of computer science, information science, and information systems. Several theoretical models have been developed and applied to study the acceptance and use behavior of information technology, of which the Technology Acceptance Model (TAM) and Unified Theory of Acceptance and Use of Technology(UTAUT) model are considered two of the most influential and widely used models by researchers [3]. They both have strong theoretical foundations that have been tested with different samples and in different situations and proved to be valid and reliable [4–6], and therefore, the present study uses them as the modeling foundation for this study of them.

Understanding the success of information systems is an area of ongoing concern not only for researchers but also for practitioners and management stakeholders. There are several ways to evaluate information systems, the most popular and effective measure is DeLone and McLean’s ISSM success model [7]. The model is considered one of the most influential theories in the field of information systems research and has been applied and validated in many studies, such as student information systems [8], e-learning systems [9], e-commerce systems [10], banking service systems [11], and others. Therefore, this study concludes that the model is a good framework for evaluating information systems.

In assessing the acceptance and influencing factors of product information systems, there are often a number of scholars who have integrated and extended the above models to achieve a better assessment, for example, Al-Adwan et al. developed an extensive model to illustrate the key factors influencing the success of e-learning systems by integrating the Information Systems Success Model (ISSM) and the Technology Acceptance Model (TAM) [12], Shinta Krisdina et al. combined the extended TAM model with enhanced care and increased accessibility and ISSM to analyze e-health acceptance [13], and scholars have also proposed an integrated framework of the TAM, UTAUT, and ISSM models [14]. Based on the proposed integrated framework of the three models, this study will also introduce two new moderating variables to study the acceptance of AI products in medical education: STARA awareness and the chilling effect.

STARA refers to the rise of smart technologies, artificial intelligence, robotics, and algorithms that are expected to lead to mass unemployment in the future [15]. It is estimated that STARA may reduce occupations by 33% by 2025 [16]. A number of scholars have found in their surveys that medical students perceive AI as a potential threat to their future careers and have developed a resistance to it [17], in addition to which some scholars have found that the majority of the population has a positive attitude that AI can be used as a diagnostic aid for physicians rather than a substitute, and suggest that AI can be incorporated into medical schools and residency training programs [18]. Since STARA may dramatically change employment dynamics and it is critical to consider its impact on medical students’ career planning, this study incorporates STARA awareness into the research model to investigate the factors influencing the use of AI products by medical students and to explore the moderating effect of this variable on the use of AI in medical education [19].

The chilling effect refers to people’s fear of publicity or indifference to public affairs due to repression, fear of punishment, or feelings of surveillance. This concept has been frequently applied in the context of new media research, as well as the emerging areas of AI and internet regulations, where it has led to increased innovation in fields such as social media, university education, and social sciences [20, 21]. Recent research has shown that artificial intelligence (AI) learning in educational settings is accompanied by increasingly intrusive monitoring of students. Some scholars have also evaluated the effectiveness of machine learning algorithms in monitoring students’ academic progress, proposing the development of new intelligent educational monitoring systems [22]. However, the pervasiveness of such monitoring environments elicits a variety of responses from students [23]and may trigger a chilling effect, which studies have shown to influence behavioral intentions in the context of new media and information [27]. However, it remains unclear whether the chilling effect exists in medical education and whether it affects medical students’ acceptance of AI technologies. Therefore, further research on the moderating role of the chilling effect at the intersection of AI and medical education is of theoretical importance.

### Significance of the study

It is expected that Artificial Intelligence will have a globally transformative impact on the economics and structure of healthcare. Consequently, medical education will encounter the potential and challenges posed by the emerging technologies of artificial intelligence. In this context, medical students, the future drivers of intelligence in medicine, will play a pivotal role in leveraging technological enhancements to propel healthcare forward. Wartman argues that focusing on how to successfully educate medical students to navigate healthcare environments altered by AI applications should be a key consideration in current curricular reforms [24]. Scholars have emphasized that the potential impact of artificial intelligence on medicine is substantial and far-reaching. They highlight that one of the biggest challenges in medical education lies in helping medical students master these new technologies and in teaching them how to make informed clinical decisions [25]. Consequently, it is widely believed that steps should be taken to incorporate AI into medical school curricula to prepare physicians for the age of AI [26].

However, despite the increasing use of AI in a rapidly changing digital environment, medical education has not kept pace with the rapid development of AI. The increasingly sophisticated use of machine learning poses a significant challenge to current models of medical education. Despite this, only a limited number of studies have examined the current status or influencing factors of medical students’ adoption of AI, highlighting the need to explore how to better integrate AI into medical education and fully leverage the potential of big data, which remains an important and underexplored topic [27]. Under these circumstances, understanding he factors influencing medical students’ ongoing willingness to use AI products is essential for enhancing the quality of these products and realizing their maximum potential benefits in medical education. This understanding is also critical for the successful introduction of AI products into the healthcare field, thereby driving advancements in big data and technology [28].

### Aim of the study

This study employs the Technology Acceptance and Information System Success model to investigate the factors influencing the acceptance of AI products in medical education. It focuses on the quality of the AI technology, as well as the chilling effect and STARA awareness. The findings of this study will serve as a valuable reference for the development of AI products in education and contribute towards the advancement of AI applications in medicine. Furthermore, it aims to maximize the use of AI in medical education make the Most of the Functions and Roles of Big Data Languages, and propel the intellectualization of medical education.

## Method

### Research model design

**Fig. 1:**
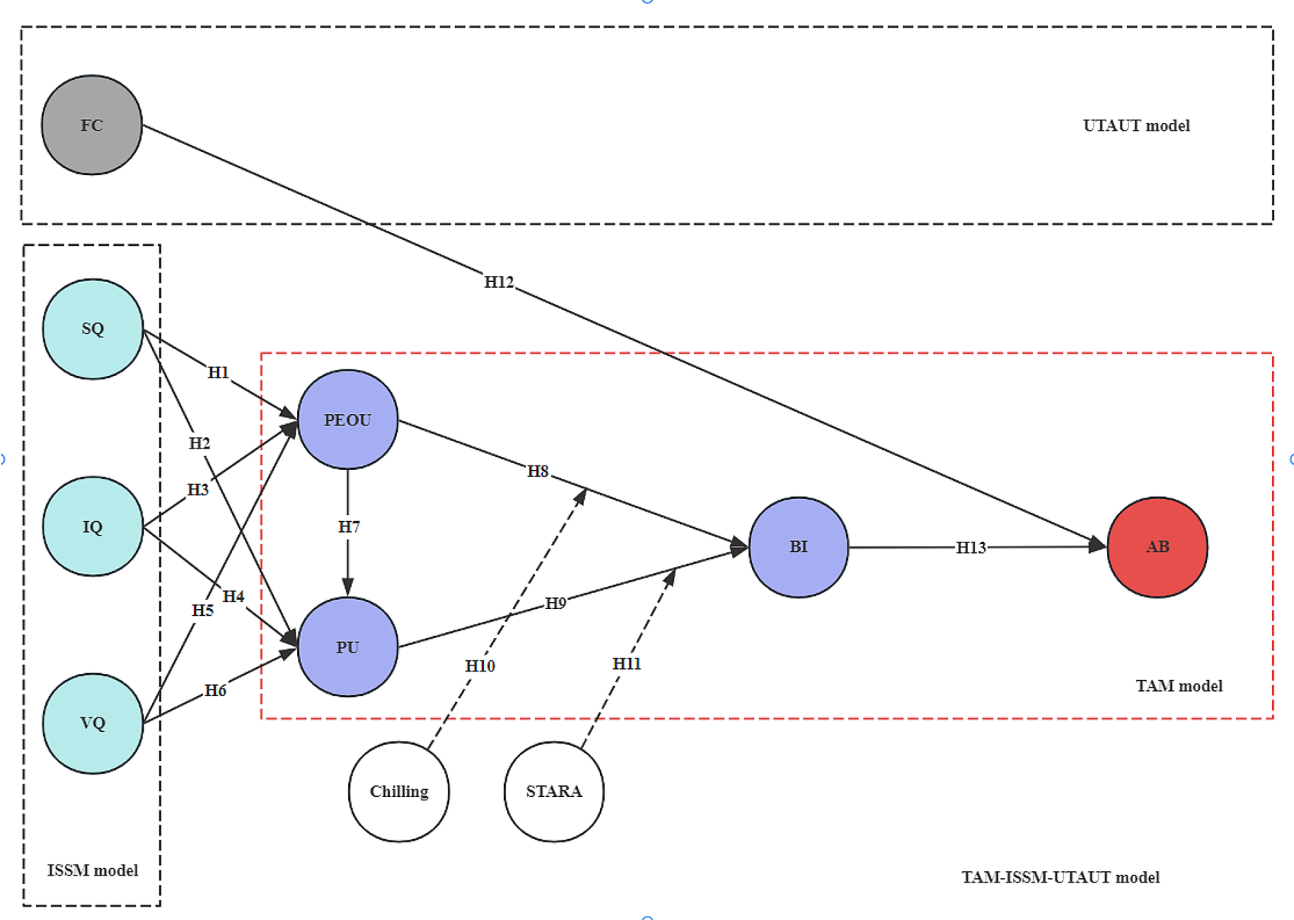
Extended TAM-ISSM-UTAUT integration model

The model architecture proposed in this study integrates TAM, ISSM, and UTAUT, and although such integration is similar to some previous studies, this study adopts the expanded TAM-ISSM-UTAUT integrated model is based on that combination and introduces the two moderating variables of the chilling effect and the STARA awareness as this study’s innovation of the theoretical foundation, the model framework is shown in Fig. 1.

#### ISSM model

System quality (SQ) refers to a user-friendly arrangement of the physical attributes of a government Web site (e.g., usability, site design, navigation, and operating modules [29]). Information quality (IQ) refers to the content displayed on any user interface of a system-produced application or online screen [30], or the ability to provide government websites with timely, accurate, comprehensive, concise, and relevant information based on citizens’ needs [31]. Service quality (VQ) is based on the assessment of the performance of information system providers, including responsiveness, assurance, and friendliness [7] .

In studying the factors influencing the perceived ease of use of information technology, Mustapha and Obid found a direct positive correlation between VQ and PEOU [32]. Similar results were found by Bahari et al. and Li, Y et al. [33]. Others have found that IQ has a positive effect on PEOU [34]. M. Yaşlıoğlu et al. have argued that PEOU is both a direct antecedent of and a direct result of VQ [35].

As for the research on the influencing factors of the perceived usefulness of information technology, some scholars have pointed out that both SQ and VQ have a significant positive effect on consumers’ PU [12].In an e-commerce study, Putri and Pujani also demonstrated the effect of SQ, IQ, and VQ on PU [36]. It has also been argued that SQ and IQ affect the PEOU of the system and also affect the PU of the system [37], and that both IQ and SQ are important exogenous predictors of BI [38].

Based on the above findings, this study infers that system quality (SQ), information quality (IQ), and service quality (VQ) are three significant predictors of perceived ease of use (PEOU) and perceived usefulness (PU) [39], and proposes the following reasonable hypotheses:

##### H1

The system quality of AI products has a significant impact on the perceived ease of use by medical students.

##### H2

The system quality of AI products has a significant impact on the perceived usefulness of medical students.

##### H3

The information quality of AI products has a significant impact on the perceived ease of use for medical students.

##### H4

The information quality of AI products has a significant impact on the perceived usefulness of medical students.

##### H5

The quality of service of AI products has a significant impact on the perceived ease of use for medical students.

##### H6

The service quality of AI products has a significant impact on the perceived usefulness of medical students.

#### TAM model

In the technology acceptance model, perceived ease of use and perceived usefulness are considered as two key factors influencing the acceptance of new technologies [40].In this case, the intention to continue using is the ending variable of the model, which refers to the customer’s final decision to purchase the product or service again based on a positive experience while using the product or service [41]. Perceived ease of use (PEOU) has been defined as the degree to which a person believes that using a particular system in an organizational setting can be done without effort or people’s impression of how much effort is required to learn a new technology or product that can be used [42], perceived usefulness (PU) is the subjective belief of users that utilizing a particular technology will improve the quality of their work [43](Majumder et al., 2022). Most of the studies have proved that PEOU has a positive effect on consumers’ behavioral intention to adopt technology [44, 45], and PU positively affects individuals’ behavioral intention to adopt technology [6, 46, 47]. Furthermore, PEOU is often recognized as an important predictor of attitudes and PU in technology acceptance studies in many contexts and cultures [48, 49].A large number of previous studies have confirmed that behavioral intention (BI) has a significant effect on actual behavior [50, 51].

Based on the above theories, this study proposes the following hypotheses:

##### H7

Perceived ease of use has a significant positive impact on medical students’ perceived usefulness.

##### H8

Perceived ease of use has a significant positive impact on medical students’ intention to continue using AI products.

##### H9

Perceived usefulness has a significant positive impact on medical students’ intention to continue using AI products.

##### H13

Intention to continue using AI products has a significant positive impact on medical students’ actual behaviour to continue using AI products.

#### UTAUT model

Facilitating conditions (FC) are defined as “the extent to which individuals perceive the technical and organizational infrastructure to support the use of the system [52]. Rachmawati et al. state that behavioral intentions and facilitating conditions have a direct impact on actual behaviors [53, 54]. A study of an academic tool MOOC also demonstrated the predictive effect of FC on AB [55]. Therefore, we propose the hypothesis:

##### H12

Facilitating conditions has a significant impact on medical students’ actual behaviour to continue using AI products.

#### Chilling effect

According to Penny and Marder et al.‘s definition of the chilling effect, it can be understood as “the effect of audience monitoring on constrained behavior [56]”. Based on previous research, Stubenvoll et al. translated chilling effect-related behaviors into reduced online search behavior, online expression of opinions, and use of websites, news, and social media [57]. A study by Ben Marder et al. revealed for the first time the potentially far-reaching implications of online peer-to-peer surveillance, revealing the importance of further research into this phenomenon [56].To understand whether the threat of such surveillance on websites related to AI products affects users’ continued willingness to use them, this study expands on the original model by focusing on the chilling effect as a new perspective that affects willingness to use.

To accurately explain it, this study defines the “chilling effect” as a situation where people are afraid to visit a specific website that collects data, make comments, etc. due to the fear of information leakage when they feel under surveillance and put forward the following hypothesis:

##### H10

The chilling effect will moderate the impact between medical students’ perceived ease of use and intention to continue using.

#### STARA awareness

New technologies may replace irreplaceable jobs, STARA may profoundly change employment, and the nature of career planning is expected to be at a critical juncture, based on these potential workplace changes, David et al. defined STARA awareness as the extent to which employees perceive the impact of smart technologies, artificial intelligence, robots, and algorithms on their future career prospects [58]. This study defines STARA awareness as the extent to which students believe their future professional lives can be replaced by these technological modalities, seven assessment items adapted from David et al. This study first tested STARA awareness to determine whether students perceived it as a threat to their future jobs/careers, and then introduced it into the expanded TAM-ISSM-UTAUT model to observe its moderating role in the effect of perceived usefulness on continuance intentions, proposing the following hypotheses:

##### H11

STARA Awareness will moderate the effect between perceived usefulness and continued willingness to use among medical students.

### Participants and procedure

**Fig. 2:**
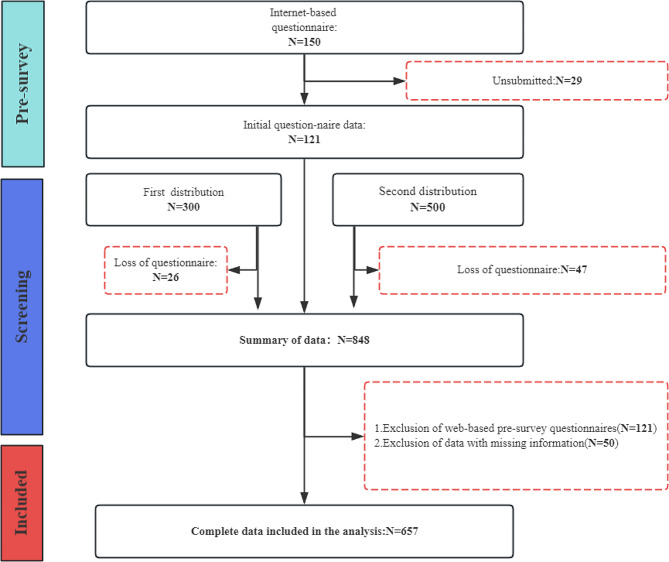
Experimental design and sample selection

Before the formal survey, this study used a web-based questionnaire platform to select 121 users for the pre-survey and modified the survey items to improve the quality of the questionnaire. The formal survey adopted an offline random sampling method, and 800 medical students were invited to conduct a paper survey at a medical university. To ensure the quality of questionnaire recovery, 300 questionnaires were allocated for the first time for data quality assessment, and the recovery rate (> 90%) was good. A total of 848 questionnaires were collected from all the preliminary surveys, and 657 valid questionnaires were finally included, with an effective rate of 77.48%, which meets the “10-fold rule” for the minimum sample size for structural equation modeling [59]. The data screening process is shown in Fig. 2. The study was approved by the Ethics Committee of Dalian Medical University. The procedures used in this study adhere to the tenets of the Declaration of Helsinki. Informed consent was obtained from all subjects and/or their legal guardians in this study. In addition, Amos software was used to avoid the effects of data distortion and to quantify the relationships between the constructs, and a validated factor analysis was used to test the reliability and validity of the measurement model. After determining the fit of the research model and data, hypothesis testing was conducted based on the results of the analysis of standardized factor loadings, path coefficients, and p-values.

**Fig. 3:**
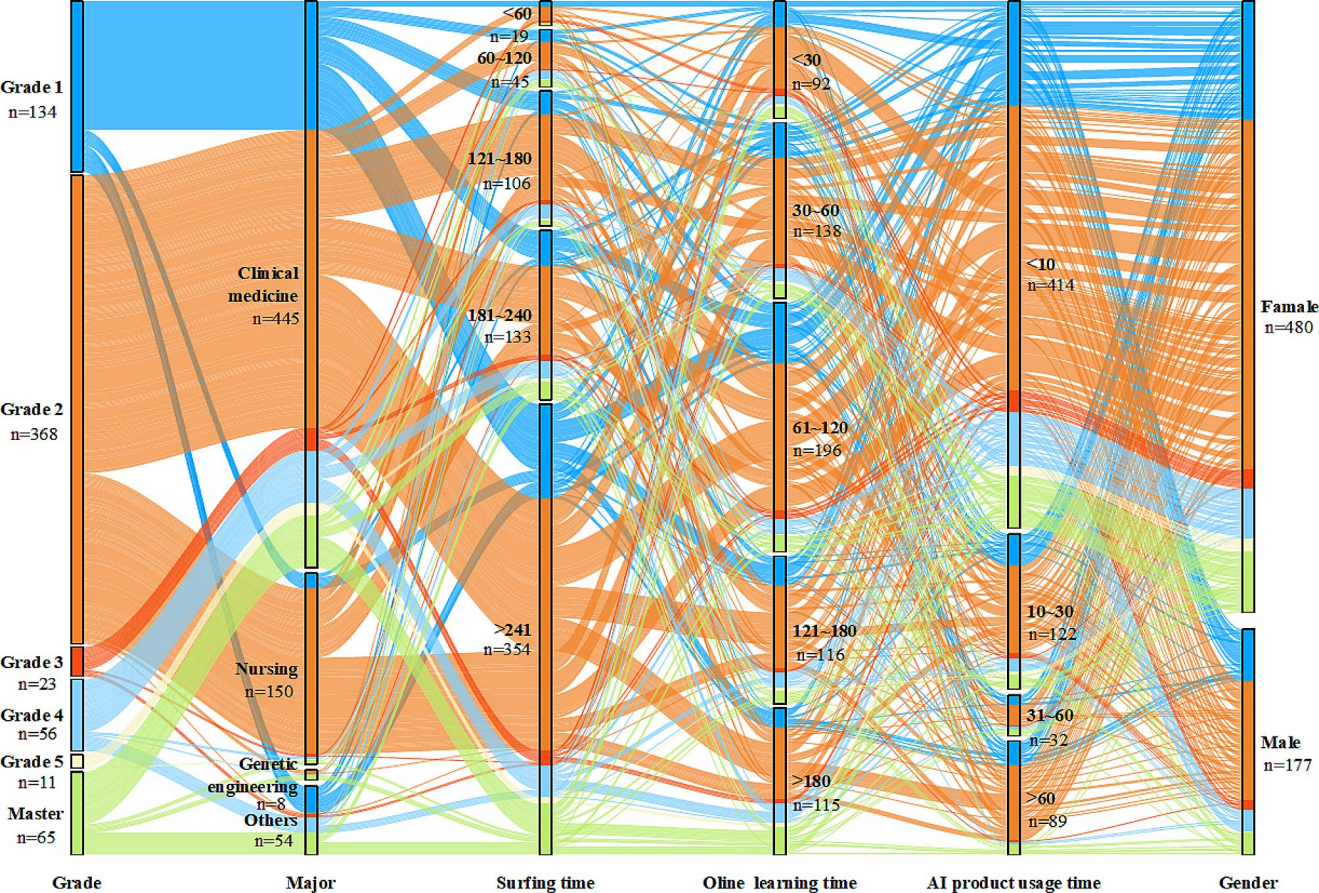
Demographic characteristics sankey diagram

This study used Amos 24.0 to analyze the data. Based on the data from the sample of 657 valid questionnaires, it was seen that the majority of the participants were female (*n* = 480,73.1%). Sophomores were the most numerous, and the majority were in medically related majors (*n* = 445,67.7%). Of these, more had used AI products (*n* = 416,63.3%), but most had used them for less than 10 min (*n* = 414,63%). The largest number of people spent 4 or more hours online (*n* = 354,53.9%), and the largest percentage of people spent 1–2 h a day studying online (*n* = 196,29.8). The majority chose that they will continue to use AI products (*n* = 545,83). Overall, the survey sample has a reasonable structure, which is conducive to the model validation of the willingness to use AI products. The characteristics of the participants are shown in Fig. 3.

### Measurements

The questionnaire items in this study were taken from the existing literature and modified appropriately according to the model constructed and the purpose of the study. The questionnaire is divided into two parts, the first part is the basic information of the respondents, including grade, gender, major, whether they have used AI products, the time they have used AI products, the time they have spent on the Internet, the time they have spent on the Internet to study, and their willingness to continue to use AI products. The second part consisted of the 10 variables and 54 measurement questions hypothesized by the study, and a questionnaire was modified based on the modeling framework Tseng, Y.‘s study [60] to determine the structure of the TAM-ISSM-UTAUT. Each question was assigned a value based on a five-point Likert scale, categorizing the options as “strongly disagree,” “disagree,” “generally,” “agree,” “strongly agree,” or “every day,” “3–5 times a week,” “1–2 times a week,” “once every two weeks,” and “once a month,” which were rated as 1, 2, 3, 4, and 5, respectively, with reverse assignments for some of the reverse dimensions, such as “STARA awareness”.

### Data analysis

The data were statistically described using SPSS version 26 and the “process” (3.3.1) macro model and Amos 23.0 software was used to build the model for this study. Users’ willingness to continue using AI products is considered an ending variable, and the rest of the variables are considered covariates, based on which this study introduces the chilling effect for moderating the effect of users’ perceived ease of use on their willingness to continue using, and STARA awareness for moderating the effect of users’ perceived value on their willingness to continue using. This study used “process” (3.3.1) macro model for moderated effects analysis, and tested for multicollinearity between variables using Pearson’s correlation test. The model’s goodness-of-fit was determined using four fit indices: relative chi-square (x2/df), Incremental Fit Index (IFI), comparative fit index (CFI), and correlated Root Mean Square Error of Approximation (RMSEA). Smaller relative chi-square values are considered to be less dependent on sample size, indicating a better fit, and values greater than 1 and less than 2 are considered good fits. The IFI and CFI range from 0 to 1, with values greater than 0.75 indicating a good fit. RMSEA is an indicator of how closely the validation structure approximates the modeled data, with values less than 0.08 indicating a good model fit. A p-value of < 0.05 was considered statistically significant.

## Results

### Measurement model assessment

The external model tests reliability, overall validity, and discriminant validity. It requires standardized factor loadings greater than 5 for each dimension. If an item’s factor loadings do not meet If an item’s factor loadings do not meet the recommended values, it is an indication that the item is not representative and, therefore, the item must be deleted. The factor loadings for all aspects of this study were greater than 0.5, so the items were retained. Cronbach’s alpha and reliability of the latent variable components were used in this study to assess the internal consistency of the dimensions. Typically, Cronbach’s alpha should be at least 0.7, and the higher the CR (Construct Reliability) of the latent variable, the higher the correlation of each item on the surface. However, Hair et al. and Fornell and Larcker recommend a CR of 0.6 or higher. The Cronbach’s alpha and CR values for all aspects of this study are higher than the Cronbach’s alpha and CR values, indicating that the model has good internal consistency, the test results are shown in Table 1. Convergent validity is used to measure the degree of convergence or correlation between multiple indicators of the same dimension. According to the relevant literature, the factor loadings for each aspect must be greater than 0.7, the constitutive reliability requirement must be greater than 0.6, and the mean extracted variance must be greater than 0.5.

**Table 1:** Combined reliability and validity analysis

Facet	Item	Parameter significance estimation				Factor loading	Composition validity	Convergence validity
		Unstd	S.E.	C.R.	P	Std	CR	AVE
SQ	v1	1.000				0.710	0.866	0.564
	v2	1.218	0.070	17.370	***	0.748		
	v3	1.228	0.068	18.175	***	0.788		
	v4	1.205	0.066	18.258	***	0.793		
	v5	1.081	0.065	16.601	***	0.712		
IQ	v6	1.000				0.790	0.842	0.475
	v7	0.882	0.049	18.171	***	0.715		
	v8	0.748	0.045	16.548	***	0.657		
	v9	0.823	0.044	18.679	***	0.733		
	v10	0.932	0.052	17.998	***	0.709		
	v11	0.692	0.057	12.092	***	0.492		
VQ	v12	1.000				0.807	0.881	0.553
	v13	1.027	0.048	21.238	***	0.778		
	v14	0.967	0.047	20.396	***	0.753		
	v15	0.952	0.047	20.242	***	0.748		
	v16	0.839	0.046	18.179	***	0.685		
	v17	0.877	0.049	18.068	***	0.681		
SA	v18	1.000				0.727	0.927	0.646
	v19	1.164	0.054	21.546	***	0.844		
	v20	1.204	0.054	22.172	***	0.868		
	v21	1.212	0.055	22.049	***	0.863		
	v22	1.142	0.053	21.368	***	0.837		
	v23	1.090	0.055	19.913	***	0.783		
	v24	0.929	0.054	17.320	***	0.685		
CE	v25	1.000				0.767	0.940	0.723
	v26	1.133	0.046	24.740	***	0.876		
	v27	1.179	0.046	25.820	***	0.907		
	v28	1.173	0.045	26.036	***	0.913		
	v29	1.060	0.047	22.549	***	0.814		
	v30	1.088	0.048	22.566	***	0.814		
BI	v31	1.000				0.878	0.884	0.533
	v32	0.940	0.031	29.995	***	0.871		
	v33	0.837	0.034	24.924	***	0.783		
	v34	0.797	0.031	25.922	***	0.801		
	v35	0.543	0.049	10.989	***	0.420		
	v36	0.722	0.040	18.193	***	0.634		
	v37	0.715	0.042	17.197	***	0.608		
FC	v38	1.000				0.682	0.699	0.384
	v39	1.007	0.082	12.307	***	0.706		
	v40	0.993	0.081	12.265	***	0.682		
	v41	0.552	0.080	6.885	***	0.325		
AB	v42	1.000				0.964	0.703	0.571
	v43	1.012	0.261	3.883	***	0.462		
PEOU	v44	1.000				0.717	0.659	0.290
	v45	0.317	0.057	5.535	***	0.245		
	v46	0.938	0.065	14.393	***	0.687		
	v47	0.250	0.065	3.856	***	0.170		
	v48	0.399	0.060	6.617	***	0.294		
	v49	0.967	0.064	15.117	***	0.759		
PU	v50	1.000				0.740	0.858	0.485
	v51	0.964	0.049	19.755	***	0.786		
	v52	1.026	0.052	19.677	***	0.783		
	v53	1.032	0.052	19.841	***	0.789		
	v54	0.954	0.058	16.437	***	0.659		
	v55	0.955	0.053	18.112	***	0.723		
	v56	0.346	0.076	4.531	***	0.186		

Note: ***p < 0.001

### Correlation analysis

The degree of differentiation indicates the degree of difference between the facets of the model; if the degree of difference between the facets is greater, then the degree of correlation between the facets is lower, which means that the facets are more differentiated from each other. According to the validity evaluation method, when the square root of the AVE on the diagonal is essentially greater than the correlation coefficient with the other factors, it indicates that the requirements of the validity criterion are met. Before making an assessment of the structural equation model, this study used Pearson correlation analysis to check for covariance to confirm that the regression results were unbiased. The results of the analysis showed (Table 2) that the absolute value of the correlation coefficient between two and two of the research variables included in this study is less than 0.8, which indicates that the probability of covariance between the variables in the model is small and has a small impact on the regression results of the model, therefore, it was chosen to include all the questionnaire variables in the constructed TAM-ISSM-UTAUT model.

**Table 2:** Correlation analysis

variables	AVE	PE	PU	SQ	IQ	VQ	SA	CE	BI	FC	AB
PE	0.290	0.538									
PU	0.485	0.380**	0.696								
SQ	0.564	0.368**	0.573**	0.751							
IQ	0.475	0.342**	0.598**	0.734**	0.689						
VQ	0.553	0.377**	0.584**	0.720**	0.733**	0.743					
SA	0.646	0.188**	-0.021	− 0.164**	− 0.129**	− 0.165**	0.804				
CE	0.723	0.05	0.095*	0.082*	0.097*	0.094*	-0.049*	0.850			
BI	0.533	0.327**	0.599**	0.479**	0.543**	0.527**	-0.073	0.159**	0.730		
FC	0.384	0.507**	0.628**	0.633**	0.717**	0.685**	-0.051**	0.084*	0.566**	0.619	
AB	0.571	0.169**	0.168**	0.091*	0.111**	0.147**	-0.003**	-0.029	0.237**	0.127**	0.756

* Significant correlation at the 0.05 level (two-tailed)

### Structural model assessment

**Fig. 4:**
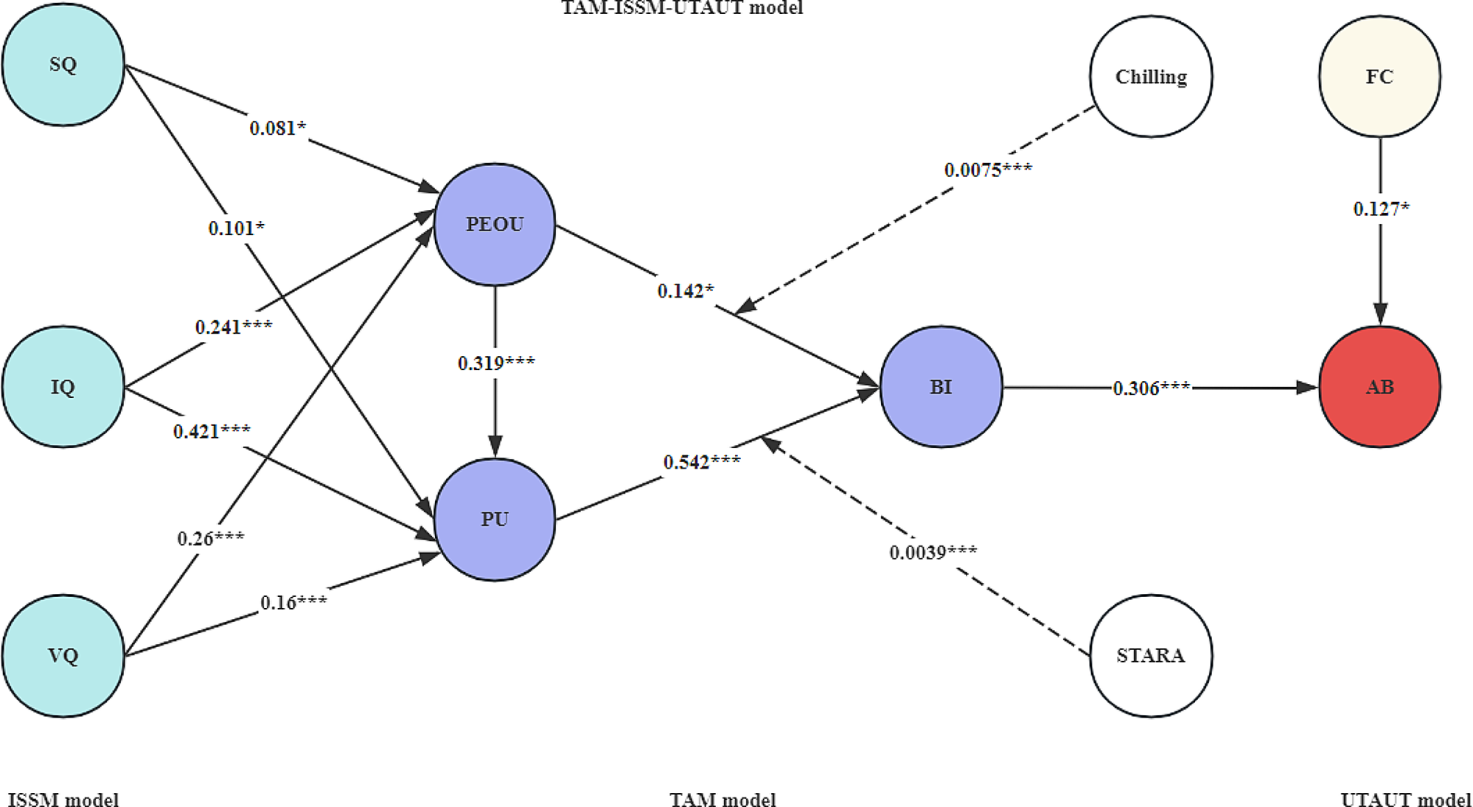
Results of TAM-ISSM-UTAUT model. (CMIN/df = 4.175, IFI = 0.795, CFI = 0.794, RMSEA = 0.07)

The structural equation model in Fig. 1 was analyzed using AMOS software to derive standardized path coefficients (Fig. 4). Structural equations have different fit metrics that can be used to measure the degree of fit between the questionnaire data and the structural equation model. Typically, the fit metrics CMIN/df is less than 5, CFI is less than 5, CFI, GFI, IFI, and NFI are all greater than or equal to 0.7, and RMSEA is less than 0.1, which indicates that the fit between the questionnaire data and the structural equation modeling is acceptable. The results of the initial model run parameters for this study are shown in Table 2. It can be seen that the fit indicators of the model are all acceptable or good (CMIN/df = 4.175, IFI = 0.795, CFI = 0.794, RMSEA = 0.07), so it can be assumed that the constructed structural equation model fits the actual questionnaire data to a high degree and the obtained data better explains the relationship between the variables within the model.

In particular, Table 2 shows that AI products SQ, IQ, and VQ have a significant positive effect on PE and PV, PE has a significant positive effect on perceived value PU, and both PE and PU have a significant positive effect on BI. BI has a significant positive effect on AB. The main path hypotheses H1, H2, H3, H4, H5, H6, H7, H8, H9, H12 and H13 were supported by the tests (see Table 3). The hypothesis of the mediating effect of the chilling effect and STARA awareness in the model did not pass the test, *p* > 0.05, not statistically significant, indicating that the mediating effect does not exist, therefore, this study used Process software for the moderating effect analysis, and the results of the analysis of Amos software are shown in Table 3; Fig. 4.

**Table 3:** Table of path coefficients

Path			Non-standardized path	Standardized path	S.E.	C.*R*.	*P*	Conclusion
PEOU	<---	SQ	0.097	0.081	0.054	1.783	0.025	support
PEOU	<---	IQ	0.25	0.241	0.049	5.102	***	support
PEOU	<---	VQ	0.275	0.26	0.049	5.575	***	support
PU	<---	SQ	0.097	0.101	0.036	2.695	0.007	support
PU	<---	IQ	0.353	0.421	0.037	9.67	***	support
PU	<---	VQ	0.136	0.16	0.033	4.09	***	support
PU	<---	PEOU	0.258	0.319	0.038	6.868	***	support
CE	<---	PEOU	0.097	0.063	0.07	1.4	0.161	Not-support
SA	<---	PU	0.059	0.045	0.056	1.062	0.288	Not-support
BI	<---	PEOU	0.151	0.142	0.048	3.15	0.002	support
BI	<---	PU	0.713	0.542	0.062	11.443	***	support
BI	<---	CE	0.078	0.113	0.024	3.278	0.001	support
BI	<---	SA	-0.061	-0.061	0.035	-1.755	0.079	Not-support
AB	<---	BI	0.197	0.306	0.026	7.474	***	support
AB	<---	FC	0.139	0.127	0.042	3.309	0.01	support

Note: ***p < 0.001

### Analysis of moderating effects

**Fig. 5:**
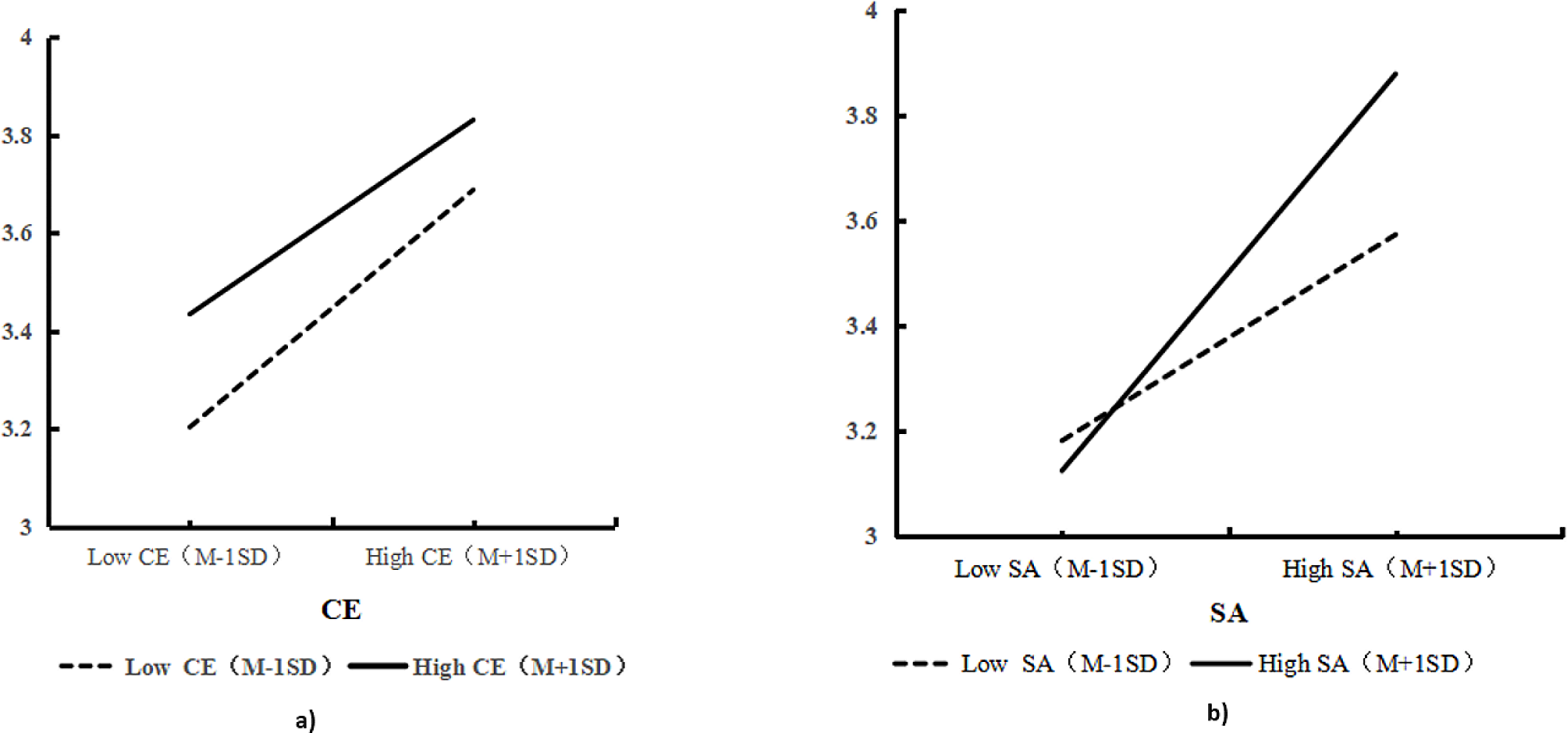
(**a**) Mediation effect test results of CE. Fig. 5**b** Mediation effect test results of SA

Based on H10 and H11, this study divided the high chilling effect group and low chilling effect group, and the high STARA-awareness group and low STARA-awareness group according to the mean plus or minus one standard deviation, and plotted the intuitive slopes as shown in Fig. 5a and b This was done to demonstrate the interaction of perceived ease of use with the chilling effect and perceived usefulness with STARA awareness. The standard errors with 95% confidence intervals obtained are shown in Table 4, and the results indicate that the interaction term of medical students’ perceived ease of use and the chilling effect has a significant effect on the willingness to continue to use the AI product (β = 0.0075, *p* < 0.05), the interaction terms of medical students’ perceived usefulness and STARA awareness also had a significant effect on the willingness to continue using the AI product (β = 0.0039, *p* < 0.05). Therefore, H10 and H11 are supported.

**Table 4:** Moderating effects

Variables	S.E.	95%BI	
PEOU→CE→BI	0.0075	-0.0039	0.0257
PU→SA→BI	0.0039	-0.0055	0.0104

## Discussion

### Optimizing medical education for the age of artificial intelligence

It is becoming increasingly important for medical education to prepare students to utilize AI technology and to be willing to adopt it in their future practice, as artificial intelligence (AI) can be considered a crucial element in shaping the future physician’s identity [61]. The results of this study show that medical students’ willingness to use AI products positively predicts their actual use behavior (H13), which is consistent with the results of a large number of previous studies [50, 51]. However, the findings of this study also showed low actual use of AI products by medical students, meaning that AI has not yet been widely integrated into medical education. One possible explanation for this could be the existing gap between developers and end-users in the field of AI applications in medical education [62]. Additionally, the application of AI products in medical education is still in the developmental stage, and some medical students are not familiar with or do not fully grasp the content of AI products. Furthermore, there has been a lack of frequent utilization of AI products as life assistants. It is essential to provide guidance for developers to create higher-quality AI products and simultaneously optimize medical education for the AI era. This includes implementing changes that strengthen theoretical guidance for medical education in line with the rapid advances in AI, ensuring that students are adequately prepared for the technologically advanced future of healthcare [63].

The results of this study showed that the convenience conditions of AI products have a significant effect on the actual use behavior of medical students (H12), consistent with results confirmed by previous researchers [64]. Therefore, both academia and medical education institutions must consider investing resources in AI training to ensure the appropriate use of the technology [65]. Future studies can further enrich the findings of this study by increasing the sample based on the framework of this study, taking into account the attributes of the use of AI products as well as the penetration of AI products in medical education in China.

### Focus on the critical role of information quality and perceived usefulness

The study showed that SQ, IQ, and VQ all impact on PU and PEOU of BI(H1, H2, H3, H4, H5, H6), consistent with Lee et al.‘s findings [39]. It was found that the quality of AI product information has the most significant effect on PU and the greatest effect on PEOU. Additionally, the study revealed that PEOU and PU of AI products for medical students significantly positively affected BI(H8, H9) [66, 67]. The positive effect of PEOU on PU was also statistically significant(H7), and PU had a greater effect on the acceptance of AI products than PEOU, aligning with S. Wangpipatwong et al.‘s findings [12]. PEOU was further confirmed to have a positive effect on PU [68], leading to the conclusion that IQ and PU are key predictors of AI technology acceptance among medical students. Previous studies [69] found that SQ, IQ, and VQ had a positive effect on PEOU and PU, with IQ having the most significant effect.

Developing efficient and practical applications is a difficult task. The results of this study provide empirical evidence to analyze the system, information, and service quality of smart products, and will help new entrant developers in the existing market to design AI products that are more suitable for medical education. This adds depth to our understanding of the dimensions of quality of smart products and enhances the theoretical understanding of the factors contributing to the perceived quality of smart products by citizens of countries whose development has not yet reached the level of the most developed countries. Medical students seek relevant, accurate, and reliable information to enrich their medical knowledge, thus the higher quality of information output encourages their use of AI products in their studies. Despite the respondents’ overall receptiveness to the new technology, concerns about cybersecurity and accuracy still exist. Efficient, user-friendly design is key to product success, developers should integrate AI principles with medical expertise to facilitate the inclusion of AI products in formal medical education, ensuring fast and effective access to information. Additionally, the integration of heterogeneous distributed information systems is crucial to improving product flexibility and simplifying the use process, aiming to provide useful and relevant information to medical students.

### Moderating effect of the chilling effect

In previous research, the impact of a product’s quality on mass acceptance has been extensively explored. However, Li J et al. have contended that consumers’ primary concern with new technologies or systems is the security of the system and the protection of their privacy [70]. Additionally, Some scholars have currently found that privacy fatigue is a significant predictor of individuals’ self-reported privacy-preserving behaviors, while chilling effects play a key role in predicting individuals’ self-reported privacy-protective behaviors [57]. Moreover, past studies have demonstrated that surveillance behaviors can lead to a chilling effect, discouraging individuals from participating in certain activities [20]. Despite the critical importance of these chilling effects of data surveillance in a digital society, there have been only scattered theoretical developments, and at most a handful of studies that have attempted to assess chilling effects in the context of data surveillance. In a pioneering move within the field of AI, our study has unearthed a similar “chilling effect” experienced by medical students when using AI products, which positively moderates their willingness to continue using AI products based on their perceived ease of use. This study illuminates the potentially profound impact of online surveillance monitoring of AI products on product acceptance and underscores the importance of further research on this phenomenon. To ensure the optimal utility of AI-based technologies in medical education, AI engineers should consider the chilling effect that AI may have on medical students more carefully in their research and development efforts, enhance the security and privacy protection of users when they utilize the Platform for information retrieval, website browsing, and content interaction, thereby appropriately controlling the triggering of the chilling effect.

### Moderating effects of STARA awareness

Brougham and Haar conducted a study in New Zealand involving 120 employees, who were assessed using the STARA Awareness Scale. The findings of the study indicated that participants exhibited minimal concern regarding the possibility of job replacement. Conversely, extensive research has been conducted on the perceptions of medical students regarding the impact of AI in various countries including Canada, Europe, and the U.S. Christianh and Pinto et al. reported that a significant proportion of medical students expressed apprehension regarding the potential implications of AI on their future practice [71]. Furthermore, a survey encompassing medical students from 19 universities in the UK highlighted that nearly half of the respondents voiced concerns about job displacement as a result of AI [72]. Similarly, a survey of Canadian medical students by B Gong et al. revealed that almost half of the participants experienced anxiety concerning the prospective career-related ramifications of emerging technologies, such as artificial intelligence [73].

The impact of artificial intelligence (AI) in medicine on medical students has been confirmed by various studies. Despite the well-researched topic of STARA (Social, Trustworthy, Alive, Responsive, and Automated) awareness, only limited research has been conducted on its integration into AI products within the field of medical education. The findings of this study further substantiate the hypothesis that STARA awareness plays a moderating role in the relationship between medical students’ perceived value and their intention to continue using AI technologies. Medical students’ perceptions of the impact of STARA technology on the future healthcare workplace are influenced by their level of STARA awareness. The introduction of new technologies such as STARA is often perceived as a potential threat to future job stability, leading to increased levels of insecurity among medical students. This heightened concern may be attributed to the increasingly prominent impact of automation technology on careers due to the rapid advancement of technology in recent years. While it is not anticipated that AI will entirely replace the role of doctors, it is expected to assume many of the tasks traditionally performed by medical professionals, thereby improving healthcare services at a faster rate. As a result, there is a growing need to establish new responsibilities and learning requirements for medical students in order to reshape their future professional identity. The fear of being replaced by a robot is another important factor to consider when designing HCI [74]. Therefore, in order to increase the willingness of medical students to use AI products, in addition to product quality and safety parameters, other parameters such as substitutability should be considered. Directly or indirectly, the willingness of medical students to use AI can be increased by increasing their identification with it. We recommend that developers influence medical students by creating psychological interventions to reduce their internal fears and increase the chances of medical students succeeding with robots in their future work environments.

## Conclusion

### Implications

The results of this study carry several important theoretical implications. First and foremost, the study contributes significantly to the existing literature on AI product development in the medical field by identifying new determinants of medical students’ intention to continue using AI products. This adds to the body of knowledge in the field and advances our understanding of the factors influencing medical students’ adoption of AI products. Furthermore, the study introduces an extended TAM-ISSM-UTAUT integrated model, which includes two moderating variables – chilling effect and STARA awareness. This model offers new perspectives for investigating the factors affecting medical students’ intention to use AI products in their academic lives. It presents a novel theoretical framework for understanding the relationship between the quality of information systems and users’ continued willingness to use them. Additionally, by shedding light on the factors influencing medical students’ intention to use AI products, the study provides valuable insights into the mindset of these users. This opens up opportunities for further research to build a comprehensive understanding of the determinants of medical students’ intention to use AI products. Finally, the study addresses the call made by Ghotbi et al [19]. in a previous study for more theoretical frameworks to be incorporated into future research. This demonstrates the study’s contribution to filling theoretical gaps and advancing the theoretical foundations in the field of AI product adoption.

**Fig. 6:**
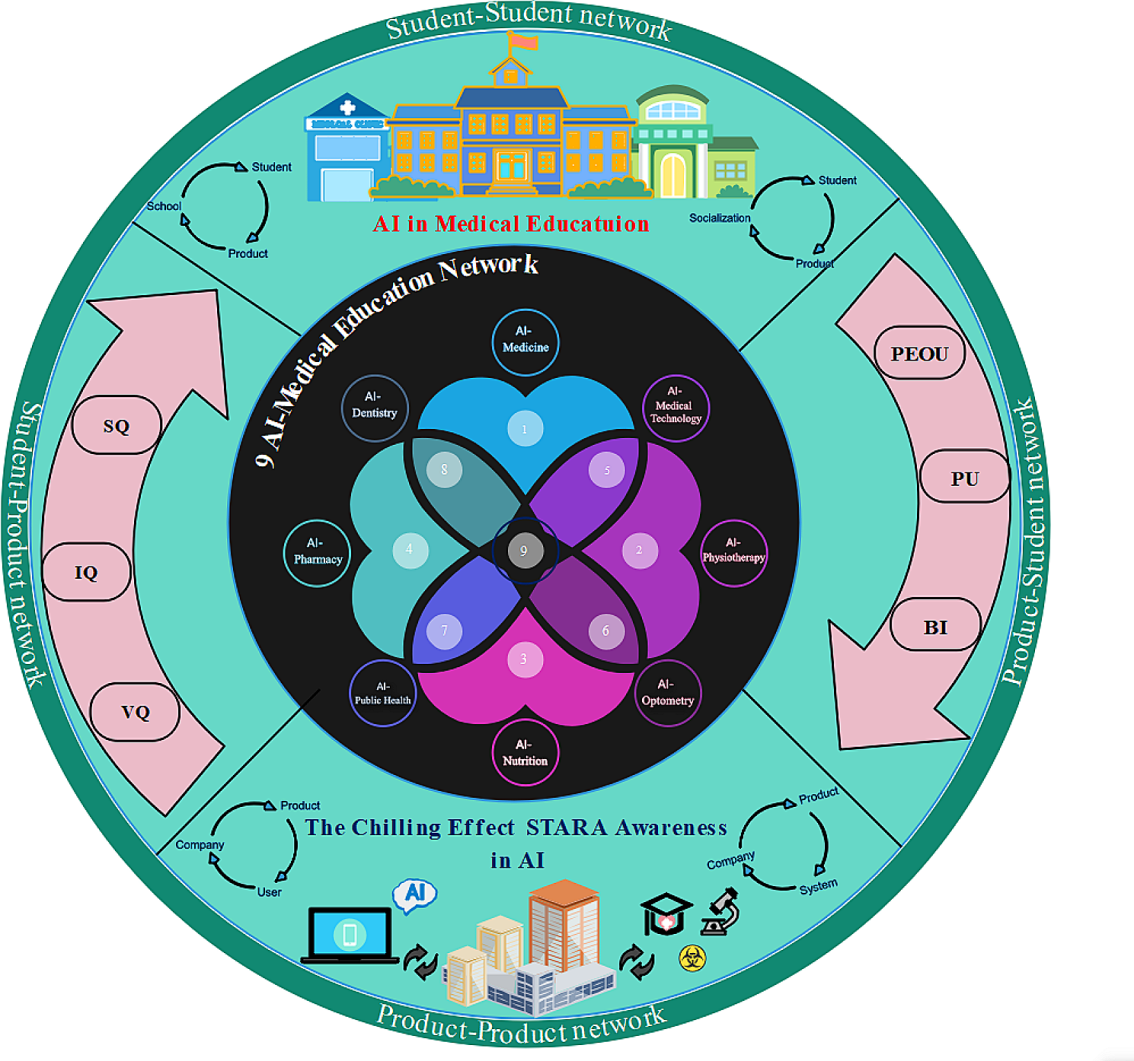
AI in medical education network

This study also provides a design concept and development framework for AI medical education products (Fig. 6), with the network framework covering three main components - “student-student network”, “product-product network " and “Student-Product Interaction Network”. The product development company mainly focuses on AI system quality, information quality, and service quality, by designing high-quality AI products to meet the user’s hardware needs for the products and increase the possibility of their application in the teaching classroom of various medical specialties. The teaching practice of AI products in different medical specialties leads to different degrees of role substitution perceived by medical students, and in general, smart technology has a higher fear level among people, so developers need to pay attention to the impact of STARA awareness on product adoption rate at the same time. At the same time, developers need to pay attention to system security and privacy protection, because the feeling of data surveillance when medical students use AI products for information retrieval, website access, and content interaction can lead them to change their digital communication behaviors, and this chilling effect is essentially a form of self-censorship in the daily use of digital media, which affects the user’s experience of interacting with the product. Users’ perceptions of various factors in the process of using a product adjust their willingness to use it mainly in the main forms of ease of use and usefulness, which are then fed back to the R&D company, constituting a closed loop of the network. The framework provides comprehensive guidance for product developers to help AI educators and researchers find and develop products that are most suitable for medical students, ensuring that current students and future doctors have enough AI exposure to advance the intelligence of products in China’s medical education sector and promoting the advancement of big data and smart technologies in society.

### Limitations and suggestions for future research

This study is the first to integrate medical education with the specification of Internet terminals. The research focused on generalizing the results by recruiting a group of medical students with similar functions to the staff for data experiments, but caution is necessary when generalizing the findings due to the specific participant group. Due to resource constraints and the shortage of AI platforms formally put into educational use, this study was unable to conduct actual testing of real scenarios during the questionnaire collection process; moreover, as AI develops and spreads, users in different countries may be affected by factors such as income differences, cultural differences, etc., and their willingness to use AI products may also be different. In the future, users from multiple countries may be selected for the study in order to compare the cognitive differences in the use of AI products among users from different countries. Although evidence on chilling effect-related behaviors varying by age, gender, or education level is limited, a recent survey suggests that chilling effects may be more pronounced in younger citizens [75], indicating the need for consideration of these differences in the present study’s findings. Few studies have quantified the chilling effect, highlighting the importance of further exploration of its dimensions in future research. The chilling effect is shown to have a temporal trajectory [76], and it is recommended that future research should employ behavioral and longitudinal data to investigate this phenomenon. Furthermore, while cross-sectional designs have been utilized to study STARA awareness, they are unable to establish the development of measurement constructs over time or causality. Thus, future research could enhance the current study by adopting a longitudinal design to examine the development of STARA awareness and job insecurity over time.

## Data Availability

The datasets used and analysed during the current study are available from the corresponding author on reasonable request.
